# F101. CANNABIS USE AND HEPATIC STEATOSIS IN PSYCHOSIS: RESULTS FROM A 3-YEAR LONGITUDINAL STUDY

**DOI:** 10.1093/schbul/sby017.632

**Published:** 2018-04-01

**Authors:** Javier Vázquez-Bourgon, Lucía Sanchez Blanco, Ruth Landera Rodriguez, Esther Setién Suero, Rodrigo Romero Jiménez, Diana Tordesillas-Gutiérrez, Rosa Ayesa Arriola, Benedicto Crespo-Facorro

**Affiliations:** 1 University Hospital Marques de Valdecilla - IDIVAL, University of Cantabria, CIBERSAM; 2 University Hospital Marqués de Valdecilla-IDIVAL; 3 Centro de Investigación Biomédica en Red de Salud Mental (CIBERSAM), Instituto de Salud Carlos III; 4 Valdecilla Biomedical Research Institute IDIVAL; 5 University Hospital Marqués de Valdecilla-IDIVAL, University of Cantabria, Centro de Investigación Biomédica en Red de Salud Mental (CIBERSAM), Instituto de Salud Carlos III

## Abstract

**Background:**

Metabolic alterations are common in patients suffering from psychosis. The rise in glycemic lipids may be related to and observed increased in the prevalence of hepatic steatosis measured by the Fatty Liver Index. However, we have recently reported a probable protective effect of cannabis smoking on weight gain and related metabolic alterations in a sample of patients drug-naïve suffering from a first episode of psychosis. We aimed to explore the effect of cannabis smoking on hepatic steatosis in a sample of first-episode non-affective psychosis patients.

**Methods:**

Anthropometric measurements, glycemic and lipid parameters, and liver steatosis index (FLI), were obtained at baseline and after 3 years of having initiated treatment. Patients were divided into two groups depending on self-reported cannabis use (cannabis users and non-users).

**Results:**

Cannabis users presented at baseline lower FLI (F=4.26, p=0.040) than non-users. These differences were also observed after 3 years of treatment (F=6.61, p=0.011).

**Discussion:**

Our results support the hypothesis that cannabis has a protective effect against hepatic steatosis. However, before being transferred to clinical practice, this study should be replicated, using larger samples.

